# 3-Nitroso-2,4,6,8-tetra­phenyl-3,7-diaza­bicyclo­[3.3.1]nonan-9-one

**DOI:** 10.1107/S1600536811022203

**Published:** 2011-06-18

**Authors:** Sampath Natarajan, Rita Mathews

**Affiliations:** a Department of Advanced Technology Fusion, Konkuk University, 1 Hwayang-dong, Gwangjin-gu, Seoul 143 701, Republic of Korea

## Abstract

In the title compound, C_31_H_27_N_3_O_2_, the two piperidine rings fused to each other each adopt a slightly distorted chair conformation. The phenyl rings on the N-unsubstituted piperidine ring occupy an equatorial position, while those on the *N*-nitroso-substituted piperidine ring are in axial positions. The NO group is approximately coplanar with the piperidine ring with a maximum deviation of 0.048 (4) Å. The dihedral angles between the mean planes of the axially and equatorially oriented phenyl rings are 27.7 (1) and 31.9 (1)°, respectively. Mol­ecular packing is stabilized by weak inter­molecular C—H⋯O and C—H⋯π inter­actions.

## Related literature

For piperidine ring conformations, see: Hofer (1976 ▶); Ramalingam *et al.* (1979 ▶); Mulekar & Berlin (1989 ▶); Pandiarajan *et al.* (1991 ▶); Rogers & Woodbrey (1962 ▶). For related structures, see: Hemalatha & Nagarajan (2010 ▶); Sampath *et al.* (2005 ▶). For puckering parameters, see: Cremer & Pople (1975 ▶). For the synthesis of the title compound, see: Noller & Baliah (1948 ▶). 
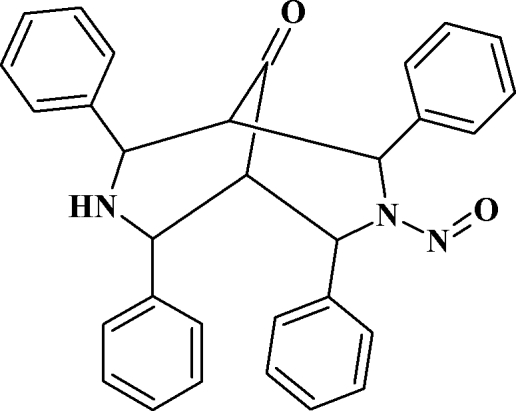


## Experimental

### 

#### Crystal data

C_31_H_27_N_3_O_2_
*M* *_r_* = 473.56
Monoclinic, 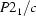

*a* = 18.723 (4) Å
*b* = 8.8319 (17) Å
*c* = 15.806 (3) Å
β = 104.728 (3)°
*V* = 2527.8 (8) Å^3^
*Z* = 4
Mo *K*α radiation
μ = 0.08 mm^−1^
*T* = 293 K
0.26 × 0.23 × 0.21 mm

#### Data collection

Bruker SMART APEX CCD diffractometer
19483 measured reflections
5385 independent reflections
3235 reflections with *I* > 2σ(*I*)
*R*_int_ = 0.056

#### Refinement

*R*[*F* ^2^ > 2σ(*F* ^2^)] = 0.081
*wR*(*F* ^2^) = 0.187
*S* = 1.06
5385 reflections
334 parameters
H-atom parameters constrained
Δρ_max_ = 0.32 e Å^−3^
Δρ_min_ = −0.29 e Å^−3^

### 

Data collection: *APEX2* (Bruker, 2004 ▶); cell refinement: *SAINT* (Bruker, 2004 ▶); data reduction: *SAINT*; program(s) used to solve structure: *SHELXS97* (Sheldrick, 2008 ▶); program(s) used to refine structure: *SHELXL97* (Sheldrick, 2008 ▶); molecular graphics: *ORTEP-3* (Farrugia, 1997 ▶); software used to prepare material for publication: *PLATON* (Spek, 2009 ▶).

## Supplementary Material

Crystal structure: contains datablock(s) I, global. DOI: 10.1107/S1600536811022203/jj2089sup1.cif

Structure factors: contains datablock(s) I. DOI: 10.1107/S1600536811022203/jj2089Isup2.hkl

Supplementary material file. DOI: 10.1107/S1600536811022203/jj2089Isup3.cml

Additional supplementary materials:  crystallographic information; 3D view; checkCIF report

## Figures and Tables

**Table 1:** Hydrogen-bond geometry (Å, °) *Cg*1 is the centroid of the C31–C36 benzene ring.

*D*—H⋯*A*	*D*—H	H⋯*A*	*D*⋯*A*	*D*—H⋯*A*
C18—H18⋯O3	0.93	2.94	3.607 (1)	130
C20—H20⋯O1	0.93	2.79	3.622 (4)	150
C24—H24⋯O2	0.93	2.64	3.276 (5)	126
C36—H36⋯O3	0.93	2.80	3.415 (9)	125
C17—H17⋯O1^i^	0.93	2.66	3.311 (4)	128
C22—H22⋯O1^ii^	0.93	2.74	3.579 (5)	150
C32—H32⋯O2^iii^	0.93	2.43	3.127 (6)	132
C34—H34⋯O3^iv^	0.93	2.24	2.878 (1)	125
C29—H29⋯*Cg*1^v^	0.93	2.87	3.677	146

Symmetry codes: (i) 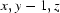; (ii) 
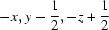
; (iii) 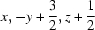; (iv) 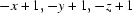; (v) 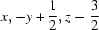.

**Fig. 1.**
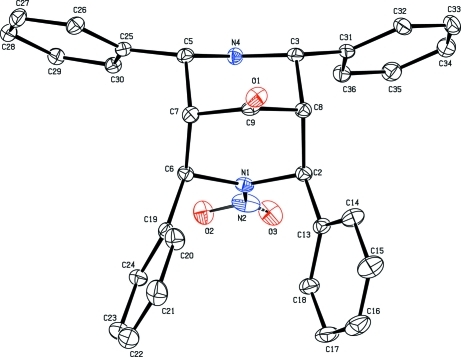
ORTEP diagram of the title molecule with the atom numbering scheme. Displacement ellipsoid are drawn at 30% probability level. H atoms were removed for clarity.

**Fig. 2.**
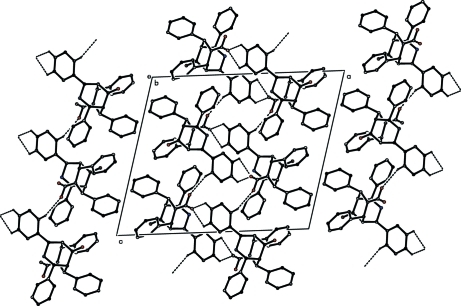
Packing diagram of the title compound viewed down the b axis. Dashed lines indicate weak C—H···O intra and intermolecular interactions.

## supplementary crystallographic information

### Comment

The piperidine ring system offers a wide variety of conformational flexibility such as chair, boat and twisted boat conformations (Hofer, 1976). However, both the chair and slightly distorted chair conformations are found to be the most favored (Ramalingam *et al.*, 1979; Mulekar & Berlin, 1989). *N*-nitroso piperidine compounds have been shown to occupy both axial and equatorial positions with the mean plane of the N—NO_2_ group being coplanar to the mean plane of the piperidine ring (Hemalatha & Nagarajan, 2010; Sampath *et al.*, 2005). The phenyl rings tend to occupy equatorial positions when the N—NO_2_ group orients itself perpendicular to the piperidine ring to avoid steric hindrance. π-electron delocalization on the N—N—O group, which restricts the free rotation of N—N bond, results in orientations that are planar (*syn*; Pandiarajan *et al.*, 1991) or perpendicular (anti; Rogers & Woodbrey, 1962) with respect to the piperidine ring. In 2,6-diphenyl-3-methyl-*N*-nitrosopiperidin- 4-one (Hemalatha & Nagarajan, 2010) the nitroso group shows both *syn* and *anti* conformations while the piperidine ring displays a boat conformation which may influence the phenyl rings to occupy axial and equitorial positions with respect to the piperidine ring.

In the title compound both piperidine rings adopt a slightly distorted chair conformation (Cremer & Pople, 1975) with puckering parameters parameters Q, θ and φ of 0.538 (3) Å, 18.0 (3)°, 142.8 (1)° [N-substituted piperidine ring (N1/C2/C8/C9/C7/C6)] and 0.657 (2) Å, 173.2 (3)° and 51.0 (2)° [N-free piperidine ring (N4/C5/C7/C9/C8/C3)], respectively (Fig. 1). For an ideal chair θ has a value of 0 or 180°. In the N-substituted piperidine ring (N1/C2/C8/C9/C7/C6) the N atom displays *sp*^2^ hybridization, as evidenced by sum of angles around the N1 atom being nearly equal to 360° [C2/N1/C6 = 122.0 (2)°, C2/N1/N2 = 123.2 (3)° and C6/N1/N2 = 114.8 (3)°]}.

Phenyl rings C13–C18 and C19–C24 are substituted axially in the N—NO_2_ piperidine ring. Torsion angles for phenyl ring C13–C18 {C6/N1/C2/C13 = 79.7 (3)°; C9/C8/C2/C13 = -69.2 (3)°} and for phenyl ring C19–C24 [C2/N1/C6/C19 = -88.8 (3)°; C9/C7/C6/C19 = 84.1 (3)°}] support this observation. The dihedral angle between these phenyl rings is 27.7 (1)°. Phenyl rings C25–C30 and C31–C36 are oriented equatorially to the piperidine ring. Torsion angles for phenyl ring C25–C30 [C3/N4/C5/C25 = 172.9 (2)°, C9/C7/C5/C25 = -171.7 (2)°] and C31–C36 [C9/C8/C3/C31 = 178.8 (2)°, C5/N4/C3/C31 = -176.2 (2)°] support this observation. The dihedral angle between these phenyl rings is 31.9 (1)°. Molecular packing is stabilized by weak C—H···O intra and intermolecular interactions and weak C—H···π intermolecular interactions (Table 1, Fig. 2).

### Experimental

Noller & Baliah (1948) developed a novel method to synthesize piperidin-4-one derivatives by the Mannich condensation reaction using respective aldehydes and ketones with ammonium acetate in the ratio of [2:1:1], respectively. The title compound was synthesized using benzaldehyde (0.2 *M*), acetone (0.1) and ammonium acetate (0.1*M*) added to pure ethanol and heated on a hot plate up to the boiling range. The resulting product of diazabicyclic[3.3.1]nonan-9-one was separated out and treated with an equimolar (1:1) quantity of NaNO_2_/HCl/80% ethanol and kept at 80° C for 4 h with vigorous stirring. The resuling title compound was separated out and crystals were grown using acetonitrile as the solvent.

### Refinement

H atoms were positioned geometrically and refined using a riding model with C—H = 0.93 Å for aromatic H, 0.97 Å for methylene, 0.96 Å for methyl H atoms and N—H = 0.86 Å. The *U*_iso_ parameters for H atoms were constraned to be 1.5*U*_eq_ of the carrier atom for the methyl H atoms and 1.2*U*_eq_ of the carrier atom for the remaining H atoms.

### Crystal data

**Table:**

C_31_H_27_N_3_O_2_	*F*(000) = 1000
*M**_r_* = 473.56	*D*_x_ = 1.244 Mg m^−^^3^
Monoclinic, *P*2_1_/*c*	Mo *K*α radiation, λ = 0.71073 Å
Hall symbol: -P 2ybc	Cell parameters from 19483 reflections
*a* = 18.723 (4) Å	θ = 2.3–27.7°
*b* = 8.8319 (17) Å	µ = 0.08 mm^−^^1^
*c* = 15.806 (3) Å	*T* = 293 K
β = 104.728 (3)°	Block, yellow
*V* = 2527.8 (8) Å^3^	0.26 × 0.23 × 0.21 mm
*Z* = 4	

### Data collection

**Table:**

Bruker SMART APEX CCD diffractometer	3235 reflections with *I* > 2σ(*I*)
Radiation source: fine-focus sealed tube	*R*_int_ = 0.056
graphite	θ_max_ = 27.7°, θ_min_ = 2.3°
ω scans	*h* = −23→21
19483 measured reflections	*k* = −11→10
5385 independent reflections	*l* = −20→20

### Refinement

**Table:**

Refinement on *F*^2^	Primary atom site location: structure-invariant direct methods
Least-squares matrix: full	Secondary atom site location: difference Fourier map
*R*[*F*^2^ > 2σ(*F*^2^)] = 0.081	Hydrogen site location: inferred from neighbouring sites
*wR*(*F*^2^) = 0.187	H-atom parameters constrained
*S* = 1.06	*w* = 1/[σ^2^(*F*_o_^2^) + (0.0715*P*)^2^ + 0.6524*P*] where *P* = (*F*_o_^2^ + 2*F*_c_^2^)/3
5385 reflections	(Δ/σ)_max_ < 0.001
334 parameters	Δρ_max_ = 0.32 e Å^−^^3^
0 restraints	Δρ_min_ = −0.29 e Å^−^^3^

### Special details

**Table:**

Geometry. All e.s.d.'s (except the e.s.d. in the dihedral angle between two l.s. planes) are estimated using the full covariance matrix. The cell e.s.d.'s are taken into account individually in the estimation of e.s.d.'s in distances, angles and torsion angles; correlations between e.s.d.'s in cell parameters are only used when they are defined by crystal symmetry. An approximate (isotropic) treatment of cell e.s.d.'s is used for estimating e.s.d.'s involving l.s. planes.
Refinement. Refinement of *F*^2^ against ALL reflections. The weighted *R*-factor *wR* and goodness of fit *S* are based on *F*^2^, conventional *R*-factors *R* are based on *F*, with *F* set to zero for negative *F*^2^. The threshold expression of *F*^2^ > σ(*F*^2^) is used only for calculating *R*-factors(gt) *etc*. and is not relevant to the choice of reflections for refinement. *R*-factors based on *F*^2^ are statistically about twice as large as those based on *F*, and *R*-factors based on ALL data will be even larger.

### Fractional atomic coordinates and isotropic or equivalent isotropic displacement parameters (Å^2^)

**Table:**

	*x*	*y*	*z*	*U*_iso_*/*U*_eq_	Occ. (<1)
O1	0.16505 (12)	0.9901 (2)	0.43401 (14)	0.0741 (6)	
O2	0.33486 (19)	0.5848 (4)	0.2575 (2)	0.0838 (10)	0.70
O3	0.3644 (5)	0.5232 (10)	0.3459 (6)	0.093 (3)	0.30
N1	0.27775 (12)	0.6798 (2)	0.34371 (15)	0.0527 (6)	
N2	0.32788 (19)	0.5919 (4)	0.3247 (3)	0.0898 (11)	
N4	0.35556 (12)	0.9558 (2)	0.38245 (13)	0.0536 (6)	
H4	0.3933	0.9259	0.3653	0.064*	
C2	0.27407 (15)	0.6783 (3)	0.43486 (18)	0.0516 (7)	
H2	0.3192	0.6290	0.4687	0.062*	
C3	0.34747 (15)	0.9322 (3)	0.47096 (17)	0.0543 (7)	
H3	0.3419	1.0322	0.4953	0.065*	
C5	0.29317 (15)	1.0350 (3)	0.32634 (18)	0.0545 (7)	
H5	0.2862	1.1294	0.3558	0.065*	
C6	0.22947 (14)	0.7796 (3)	0.27724 (17)	0.0503 (7)	
H6	0.2550	0.7961	0.2311	0.060*	
C7	0.22234 (15)	0.9354 (3)	0.31808 (18)	0.0540 (7)	
H7	0.1799	0.9887	0.2809	0.065*	
C8	0.27560 (15)	0.8422 (3)	0.46805 (18)	0.0515 (7)	
H8	0.2679	0.8404	0.5271	0.062*	
C9	0.21246 (16)	0.9267 (3)	0.40905 (19)	0.0549 (7)	
C13	0.20946 (16)	0.5843 (3)	0.44686 (19)	0.0580 (7)	
C14	0.16836 (19)	0.6217 (4)	0.5048 (2)	0.0810 (10)	
H14	0.1786	0.7108	0.5370	0.097*	
C15	0.1124 (2)	0.5289 (5)	0.5158 (3)	0.1094 (14)	
H15	0.0856	0.5562	0.5555	0.131*	
C16	0.0954 (2)	0.3983 (5)	0.4698 (4)	0.1124 (15)	
H16	0.0572	0.3367	0.4774	0.135*	
C17	0.1357 (2)	0.3594 (4)	0.4123 (3)	0.0961 (13)	
H17	0.1248	0.2701	0.3805	0.115*	
C18	0.19191 (18)	0.4497 (3)	0.4006 (2)	0.0742 (9)	
H18	0.2187	0.4207	0.3611	0.089*	
C19	0.15692 (15)	0.7006 (3)	0.23480 (17)	0.0542 (7)	
C20	0.09218 (17)	0.7261 (4)	0.2579 (2)	0.0790 (10)	
H20	0.0910	0.7970	0.3010	0.095*	
C21	0.0286 (2)	0.6478 (6)	0.2178 (3)	0.1031 (13)	
H21	−0.0146	0.6645	0.2352	0.124*	
C22	0.0291 (2)	0.5462 (5)	0.1529 (3)	0.1059 (14)	
H22	−0.0141	0.4959	0.1250	0.127*	
C23	0.0928 (2)	0.5184 (4)	0.1291 (2)	0.0902 (11)	
H23	0.0934	0.4483	0.0853	0.108*	
C24	0.15639 (18)	0.5950 (4)	0.1703 (2)	0.0703 (9)	
H24	0.1999	0.5748	0.1543	0.084*	
C25	0.30623 (15)	1.0759 (3)	0.23874 (17)	0.0511 (7)	
C26	0.26664 (17)	1.1952 (3)	0.1924 (2)	0.0643 (8)	
H26	0.2324	1.2468	0.2151	0.077*	
C27	0.27791 (18)	1.2380 (3)	0.1124 (2)	0.0707 (9)	
H27	0.2507	1.3171	0.0810	0.085*	
C28	0.32896 (18)	1.1636 (3)	0.0797 (2)	0.0661 (8)	
H28	0.3372	1.1932	0.0265	0.079*	
C29	0.36835 (16)	1.0447 (3)	0.12552 (18)	0.0597 (8)	
H29	0.4033	0.9944	0.1033	0.072*	
C30	0.35600 (15)	1.0001 (3)	0.20431 (18)	0.0562 (7)	
H30	0.3817	0.9180	0.2342	0.067*	
C31	0.41479 (15)	0.8592 (3)	0.53034 (17)	0.0544 (7)	
C32	0.42814 (19)	0.8818 (4)	0.6201 (2)	0.0715 (9)	
H32	0.3973	0.9457	0.6414	0.086*	
C33	0.4857 (2)	0.8118 (5)	0.6777 (2)	0.0869 (11)	
H33	0.4932	0.8268	0.7376	0.104*	
C34	0.53198 (19)	0.7201 (4)	0.6469 (3)	0.0856 (11)	
H34	0.5714	0.6729	0.6858	0.103*	
C35	0.52064 (18)	0.6971 (4)	0.5588 (2)	0.0773 (9)	
H35	0.5525	0.6347	0.5381	0.093*	
C36	0.46200 (17)	0.7664 (3)	0.5005 (2)	0.0653 (8)	
H36	0.4545	0.7501	0.4408	0.078*	

### Atomic displacement parameters (Å^2^)

**Table:**

	*U*^11^	*U*^22^	*U*^33^	*U*^12^	*U*^13^	*U*^23^
O1	0.0797 (15)	0.0625 (13)	0.0918 (15)	0.0167 (12)	0.0435 (12)	−0.0036 (11)
O2	0.084 (2)	0.102 (3)	0.073 (2)	0.0220 (19)	0.035 (2)	−0.008 (2)
O3	0.071 (6)	0.098 (7)	0.104 (7)	0.057 (5)	0.010 (5)	−0.026 (5)
N1	0.0479 (13)	0.0444 (13)	0.0656 (15)	0.0035 (11)	0.0142 (11)	−0.0147 (11)
N2	0.061 (2)	0.080 (2)	0.138 (3)	0.0025 (17)	0.043 (2)	−0.046 (2)
N4	0.0585 (14)	0.0537 (13)	0.0498 (13)	0.0027 (11)	0.0160 (11)	0.0018 (10)
C2	0.0512 (16)	0.0430 (15)	0.0568 (16)	0.0047 (12)	0.0065 (12)	−0.0032 (12)
C3	0.0674 (19)	0.0408 (15)	0.0549 (17)	−0.0088 (13)	0.0158 (14)	−0.0089 (12)
C5	0.0640 (18)	0.0404 (14)	0.0599 (17)	0.0002 (13)	0.0170 (14)	−0.0034 (13)
C6	0.0507 (16)	0.0524 (16)	0.0493 (15)	−0.0006 (13)	0.0153 (12)	−0.0047 (12)
C7	0.0532 (17)	0.0478 (16)	0.0607 (17)	0.0107 (13)	0.0141 (13)	0.0007 (13)
C8	0.0606 (17)	0.0444 (15)	0.0523 (16)	0.0012 (13)	0.0191 (13)	−0.0053 (12)
C9	0.0620 (18)	0.0377 (14)	0.0709 (19)	−0.0018 (13)	0.0276 (15)	−0.0084 (13)
C13	0.0533 (18)	0.0453 (16)	0.0697 (19)	0.0009 (13)	0.0050 (15)	0.0067 (14)
C14	0.075 (2)	0.068 (2)	0.108 (3)	−0.0119 (18)	0.038 (2)	0.0035 (19)
C15	0.087 (3)	0.107 (3)	0.147 (4)	−0.019 (3)	0.053 (3)	0.012 (3)
C16	0.074 (3)	0.093 (3)	0.162 (5)	−0.028 (2)	0.017 (3)	0.031 (3)
C17	0.079 (3)	0.056 (2)	0.132 (4)	−0.022 (2)	−0.012 (2)	0.009 (2)
C18	0.073 (2)	0.0497 (18)	0.089 (2)	−0.0035 (16)	0.0001 (17)	0.0031 (16)
C19	0.0469 (16)	0.0601 (17)	0.0524 (16)	−0.0047 (13)	0.0067 (13)	0.0097 (14)
C20	0.0501 (19)	0.103 (3)	0.079 (2)	0.0021 (18)	0.0086 (16)	0.004 (2)
C21	0.053 (2)	0.148 (4)	0.101 (3)	−0.005 (2)	0.006 (2)	0.023 (3)
C22	0.074 (3)	0.123 (4)	0.102 (3)	−0.043 (3)	−0.013 (2)	0.024 (3)
C23	0.086 (3)	0.089 (3)	0.083 (2)	−0.032 (2)	0.000 (2)	−0.005 (2)
C24	0.076 (2)	0.069 (2)	0.0625 (19)	−0.0207 (17)	0.0114 (16)	−0.0060 (16)
C25	0.0558 (17)	0.0409 (14)	0.0543 (16)	−0.0061 (13)	0.0098 (13)	0.0000 (12)
C26	0.071 (2)	0.0507 (17)	0.073 (2)	0.0058 (15)	0.0221 (16)	0.0036 (15)
C27	0.086 (2)	0.0535 (18)	0.072 (2)	0.0068 (17)	0.0191 (18)	0.0160 (16)
C28	0.081 (2)	0.0608 (19)	0.0581 (18)	−0.0090 (17)	0.0204 (16)	0.0047 (15)
C29	0.0651 (19)	0.0565 (18)	0.0562 (18)	−0.0012 (15)	0.0130 (14)	−0.0037 (14)
C30	0.0600 (18)	0.0480 (16)	0.0574 (17)	0.0004 (14)	0.0087 (14)	−0.0002 (13)
C31	0.0575 (18)	0.0530 (16)	0.0503 (16)	−0.0200 (14)	0.0089 (13)	−0.0020 (13)
C32	0.075 (2)	0.079 (2)	0.0576 (19)	−0.0224 (18)	0.0117 (17)	−0.0106 (16)
C33	0.080 (3)	0.113 (3)	0.056 (2)	−0.040 (2)	−0.0030 (19)	0.004 (2)
C34	0.064 (2)	0.096 (3)	0.082 (3)	−0.028 (2)	−0.0077 (19)	0.020 (2)
C35	0.061 (2)	0.078 (2)	0.088 (3)	−0.0081 (17)	0.0083 (18)	0.0080 (19)
C36	0.0650 (19)	0.068 (2)	0.0611 (18)	−0.0108 (17)	0.0129 (16)	−0.0003 (16)

### Geometric parameters (Å, °)

**Table:**

O1—C9	1.198 (3)	C17—H17	0.9300
O2—N2	1.106 (4)	C18—H18	0.9300
O2—O3	1.471 (10)	C19—C20	1.370 (4)
O3—N2	0.912 (7)	C19—C24	1.380 (4)
N1—N2	1.310 (4)	C20—C21	1.383 (5)
N1—C2	1.460 (3)	C20—H20	0.9300
N1—C6	1.488 (3)	C21—C22	1.365 (6)
N4—C5	1.454 (3)	C21—H21	0.9300
N4—C3	1.460 (3)	C22—C23	1.360 (5)
N4—H4	0.8600	C22—H22	0.9300
C2—C13	1.518 (4)	C23—C24	1.381 (4)
C2—C8	1.537 (3)	C23—H23	0.9300
C2—H2	0.9800	C24—H24	0.9300
C3—C31	1.511 (4)	C25—C30	1.368 (4)
C3—C8	1.553 (4)	C25—C26	1.385 (4)
C3—H3	0.9800	C26—C27	1.387 (4)
C5—C25	1.510 (4)	C26—H26	0.9300
C5—C7	1.569 (4)	C27—C28	1.364 (4)
C5—H5	0.9800	C27—H27	0.9300
C6—C19	1.522 (4)	C28—C29	1.378 (4)
C6—C7	1.541 (4)	C28—H28	0.9300
C6—H6	0.9800	C29—C30	1.381 (4)
C7—C9	1.498 (4)	C29—H29	0.9300
C7—H7	0.9800	C30—H30	0.9300
C8—C9	1.505 (4)	C31—C36	1.374 (4)
C8—H8	0.9800	C31—C32	1.391 (4)
C13—C14	1.378 (4)	C32—C33	1.369 (5)
C13—C18	1.390 (4)	C32—H32	0.9300
C14—C15	1.376 (5)	C33—C34	1.364 (5)
C14—H14	0.9300	C33—H33	0.9300
C15—C16	1.357 (6)	C34—C35	1.369 (5)
C15—H15	0.9300	C34—H34	0.9300
C16—C17	1.365 (6)	C35—C36	1.384 (4)
C16—H16	0.9300	C35—H35	0.9300
C17—C18	1.370 (5)	C36—H36	0.9300
			
N2—O3—O2	48.6 (5)	C16—C17—C18	121.2 (4)
N2—N1—C2	115.9 (3)	C16—C17—H17	119.4
N2—N1—C6	122.1 (3)	C18—C17—H17	119.4
C2—N1—C6	122.0 (2)	C17—C18—C13	120.9 (4)
O3—N2—O2	93.1 (7)	C17—C18—H18	119.6
O3—N2—N1	145.5 (9)	C13—C18—H18	119.6
O2—N2—N1	121.4 (5)	C20—C19—C24	117.8 (3)
C5—N4—C3	113.0 (2)	C20—C19—C6	123.9 (3)
C5—N4—H4	123.5	C24—C19—C6	118.2 (3)
C3—N4—H4	123.5	C19—C20—C21	120.9 (4)
N1—C2—C13	111.5 (2)	C19—C20—H20	119.6
N1—C2—C8	109.1 (2)	C21—C20—H20	119.6
C13—C2—C8	114.7 (2)	C22—C21—C20	120.2 (4)
N1—C2—H2	107.0	C22—C21—H21	119.9
C13—C2—H2	107.0	C20—C21—H21	119.9
C8—C2—H2	107.0	C23—C22—C21	120.1 (4)
N4—C3—C31	112.5 (2)	C23—C22—H22	120.0
N4—C3—C8	110.2 (2)	C21—C22—H22	120.0
C31—C3—C8	112.3 (2)	C22—C23—C24	119.5 (4)
N4—C3—H3	107.2	C22—C23—H23	120.3
C31—C3—H3	107.2	C24—C23—H23	120.3
C8—C3—H3	107.2	C23—C24—C19	121.6 (3)
N4—C5—C25	112.5 (2)	C23—C24—H24	119.2
N4—C5—C7	108.2 (2)	C19—C24—H24	119.2
C25—C5—C7	112.9 (2)	C30—C25—C26	119.3 (3)
N4—C5—H5	107.7	C30—C25—C5	122.2 (2)
C25—C5—H5	107.7	C26—C25—C5	118.5 (3)
C7—C5—H5	107.7	C25—C26—C27	120.3 (3)
N1—C6—C19	110.7 (2)	C25—C26—H26	119.9
N1—C6—C7	109.6 (2)	C27—C26—H26	119.9
C19—C6—C7	115.5 (2)	C28—C27—C26	119.8 (3)
N1—C6—H6	106.8	C28—C27—H27	120.1
C19—C6—H6	106.8	C26—C27—H27	120.1
C7—C6—H6	106.8	C27—C28—C29	120.1 (3)
C9—C7—C6	113.7 (2)	C27—C28—H28	120.0
C9—C7—C5	104.9 (2)	C29—C28—H28	120.0
C6—C7—C5	112.0 (2)	C28—C29—C30	120.1 (3)
C9—C7—H7	108.7	C28—C29—H29	120.0
C6—C7—H7	108.7	C30—C29—H29	120.0
C5—C7—H7	108.7	C25—C30—C29	120.4 (3)
C9—C8—C2	108.2 (2)	C25—C30—H30	119.8
C9—C8—C3	107.6 (2)	C29—C30—H30	119.8
C2—C8—C3	115.7 (2)	C36—C31—C32	118.1 (3)
C9—C8—H8	108.4	C36—C31—C3	123.3 (3)
C2—C8—H8	108.4	C32—C31—C3	118.5 (3)
C3—C8—H8	108.4	C33—C32—C31	121.4 (3)
O1—C9—C7	125.1 (3)	C33—C32—H32	119.3
O1—C9—C8	124.0 (3)	C31—C32—H32	119.3
C7—C9—C8	110.6 (2)	C34—C33—C32	119.6 (3)
C14—C13—C18	117.2 (3)	C34—C33—H33	120.2
C14—C13—C2	123.3 (3)	C32—C33—H33	120.2
C18—C13—C2	119.4 (3)	C33—C34—C35	120.2 (3)
C13—C14—C15	120.9 (4)	C33—C34—H34	119.9
C13—C14—H14	119.6	C35—C34—H34	119.9
C15—C14—H14	119.6	C34—C35—C36	120.2 (3)
C16—C15—C14	121.4 (4)	C34—C35—H35	119.9
C16—C15—H15	119.3	C36—C35—H35	119.9
C14—C15—H15	119.3	C31—C36—C35	120.4 (3)
C15—C16—C17	118.4 (4)	C31—C36—H36	119.8
C15—C16—H16	120.8	C35—C36—H36	119.8
C17—C16—H16	120.8		
			
O2—O3—N2—N1	178.8 (13)	C8—C2—C13—C18	163.9 (2)
O3—O2—N2—N1	−179.2 (9)	C18—C13—C14—C15	−0.1 (5)
C2—N1—N2—O3	1.2 (14)	C2—C13—C14—C15	−177.2 (3)
C6—N1—N2—O3	178.9 (13)	C13—C14—C15—C16	−0.4 (6)
C2—N1—N2—O2	179.8 (3)	C14—C15—C16—C17	0.5 (7)
C6—N1—N2—O2	−2.5 (5)	C15—C16—C17—C18	−0.2 (6)
N2—N1—C2—C13	−102.7 (3)	C16—C17—C18—C13	−0.2 (5)
C6—N1—C2—C13	79.6 (3)	C14—C13—C18—C17	0.4 (5)
N2—N1—C2—C8	129.5 (3)	C2—C13—C18—C17	177.6 (3)
C6—N1—C2—C8	−48.2 (3)	N1—C6—C19—C20	99.8 (3)
C5—N4—C3—C31	−176.2 (2)	C7—C6—C19—C20	−25.5 (4)
C5—N4—C3—C8	57.7 (3)	N1—C6—C19—C24	−79.0 (3)
C3—N4—C5—C25	172.9 (2)	C7—C6—C19—C24	155.7 (3)
C3—N4—C5—C7	−61.8 (3)	C24—C19—C20—C21	0.2 (5)
N2—N1—C6—C19	93.7 (3)	C6—C19—C20—C21	−178.6 (3)
C2—N1—C6—C19	−88.8 (3)	C19—C20—C21—C22	−1.6 (6)
N2—N1—C6—C7	−137.7 (3)	C20—C21—C22—C23	1.9 (6)
C2—N1—C6—C7	39.8 (3)	C21—C22—C23—C24	−0.7 (6)
N1—C6—C7—C9	−42.0 (3)	C22—C23—C24—C19	−0.7 (5)
C19—C6—C7—C9	83.9 (3)	C20—C19—C24—C23	0.9 (5)
N1—C6—C7—C5	76.7 (3)	C6—C19—C24—C23	179.8 (3)
C19—C6—C7—C5	−157.4 (2)	N4—C5—C25—C30	23.0 (3)
N4—C5—C7—C9	63.2 (3)	C7—C5—C25—C30	−99.8 (3)
C25—C5—C7—C9	−171.7 (2)	N4—C5—C25—C26	−156.5 (2)
N4—C5—C7—C6	−60.6 (3)	C7—C5—C25—C26	80.7 (3)
C25—C5—C7—C6	64.6 (3)	C30—C25—C26—C27	−0.6 (4)
N1—C2—C8—C9	56.8 (3)	C5—C25—C26—C27	178.9 (3)
C13—C2—C8—C9	−69.1 (3)	C25—C26—C27—C28	−0.9 (5)
N1—C2—C8—C3	−63.9 (3)	C26—C27—C28—C29	1.1 (5)
C13—C2—C8—C3	170.1 (2)	C27—C28—C29—C30	0.3 (4)
N4—C3—C8—C9	−55.1 (3)	C26—C25—C30—C29	2.0 (4)
C31—C3—C8—C9	178.7 (2)	C5—C25—C30—C29	−177.5 (2)
N4—C3—C8—C2	66.0 (3)	C28—C29—C30—C25	−1.8 (4)
C31—C3—C8—C2	−60.3 (3)	N4—C3—C31—C36	−27.2 (4)
C6—C7—C9—O1	−129.3 (3)	C8—C3—C31—C36	97.7 (3)
C5—C7—C9—O1	108.0 (3)	N4—C3—C31—C32	155.5 (2)
C6—C7—C9—C8	57.3 (3)	C8—C3—C31—C32	−79.5 (3)
C5—C7—C9—C8	−65.3 (3)	C36—C31—C32—C33	−1.2 (4)
C2—C8—C9—O1	122.8 (3)	C3—C31—C32—C33	176.2 (3)
C3—C8—C9—O1	−111.6 (3)	C31—C32—C33—C34	1.2 (5)
C2—C8—C9—C7	−63.8 (3)	C32—C33—C34—C35	−0.5 (5)
C3—C8—C9—C7	61.8 (3)	C33—C34—C35—C36	−0.2 (5)
N1—C2—C13—C14	−143.8 (3)	C32—C31—C36—C35	0.5 (4)
C8—C2—C13—C14	−19.1 (4)	C3—C31—C36—C35	−176.8 (3)
N1—C2—C13—C18	39.2 (3)	C34—C35—C36—C31	0.2 (5)

### Hydrogen-bond geometry (Å, °)

**Table:**

Cg1 is the centroid of the C31–C36 benzene ring.

**Table:**

*D*—H···*A*	*D*—H	H···*A*	*D*···*A*	*D*—H···*A*
C18—H18···O3	0.93	2.94	3.607 (1)	130
C20—H20···O1	0.93	2.79	3.622 (4)	150
C24—H24···O2	0.93	2.64	3.276 (5)	126
C36—H36···O3	0.93	2.80	3.415 (9)	125
C17—H17···O1^i^	0.93	2.66	3.311 (4)	128
C22—H22···O1^ii^	0.93	2.74	3.579 (5)	150
C32—H32···O2^iii^	0.93	2.43	3.127 (6)	132
C34—H34···O3^iv^	0.93	2.24	2.878 (1)	125
C29—H29···Cg1^v^	0.93	2.87	3.677	146

Symmetry codes: (i) *x*, *y*−1, *z*; (ii) −*x*, *y*−1/2, −*z*+1/2; (iii) *x*, −*y*+3/2, *z*+1/2; (iv) −*x*+1, −*y*+1, −*z*+1; (v) *x*, −*y*+1/2, *z*−3/2.
